# Risk Factors Associated with Psychiatric Hospitalization Among Iranian Schizophrenic Patients

**Published:** 2013

**Authors:** Nader Mansouri, Narges Chimeh, Mohsen Dehghani, Seyed Kazem Malakouti, Hamid Taherkhani, Zohreh Abarashi

**Affiliations:** 1 Department of Clinical Psychology, Family Research Institute, Shahid Beheshti University, Tehran, Iran.; 2 Professor, Department of Psychiatry, Family Research Institute, Shahid Beheshti University, Tehran, Iran.; 3 Professor, Department of Psychiatry, Mental Health Research Centre, Iran University of Medical Sciences, Tehran, Iran.

**Keywords:** Hospitalization, Risk Factor, Schizophrenia

## Abstract

**Objective:** Psychiatric hospitalization of patients imposes heavy burdens on caregivers, but little is known about this issue in Iran. The present cross-sectional study aimed to investigate the risk factors associated with psychiatric hospitalization of patients with schizophrenia who were the regular clients for the educational programs of The Iranian Society for Supporting Individuals with Schizophrenia (ISSIS) in Tehran, Iran.

**Methods:** 231 male and female study subjects and 231 of their caregivers participated in the study. The study subjects were independently assessed in demographics, clinical and symptom-related characteristics and basic life skills domains. Their caregivers were assessed in domains of knowledge on schizophrenia, burden, social support, family function, and the patterns of relationships with their patients and the role of health and supportive services. Data were analyzed by performing logistic regression model.

**Results: **Old age, low level of education, unemployment, greater severity of positive and negative symptoms, poor basic life skills among subjects, and objective family burden, inadequate knowledge on schizophrenia, low perceived social support and lack of medical insurance among caregivers were the most important factors associated with psychiatric hospitalization among the clients.

**Conclusions:** Some factors originated in Iranian patients and their caregivers could cause patients’ pathways to psychiatric hospitalization. Although the study results did not establish causation, based on the findings, psychoeducational interventions may reduce schizophrenia referral and lower the rate of need to inpatient services in Iran.

**Declaration of interest:** None.

## Introduction

Schizophrenia is a chronic and disabling psychiatric disorder. In addition to direct burden upon community, the lifetime, emotional, social and financial burden experienced by patients with schizophrenia impact their families. Some studies showed an estimated 50% to 80% of patients with psychotic disorders living with or having regular contact with one of their family caregivers (1) and these caregivers report crucial burden in providing care (2). 

Some risk factors can precipitate patients' pathways toward psychiatric hospitalization and these factors could impose heavy burdens on their caregivers and the community. Previous studies in developed countries show that financial, emotional and practical burdens (2), reduced social contact and lower social support (3), emotional distress (4), stigmatization (5), stressors related to patients such as positive and negative symptoms of schizophrenia and disruptive symptoms such as psychotic and aggressive symptoms, frequent hospitalizations in psychiatric wards and short length of illness (2, 6-11) are heavy burdens for caregivers and could lead to psychiatric hospitalization of patients. In Iran, an epidemiological survey of psychiatric disorders on 25,180 adults showed the annual prevalence of psychotic disorders including schizophrenia was 0.089% (12).

As a developing country, no study has been ever conducted on risk factors associated with psychiatric hospitalization among Iranian patients, while this issue has important clinical and financial implications. Iran lacks sufficient health services for psychiatric hospitalization of patients and inadequate community-based facilities, and outreach services are a critical problem. This issue has resulted in imposing the burden of care on families (13) and therefore substantially high rates of psychiatric hospitalization. 

A recent study showed Iranian caregivers experience a significant level of problem in their mental health status (14). Although a study showed that Iranian families have willingness to take care of their patient members at home and they reported their needs for availability of hospital beds at the time of referral and urgent requirements for rehabilitation and educational programs (15) in recent years, we witnessed a gradual increasing trend of psychiatric hospitalization of Iranian clients through family referrals, while underlying risk factors associated with this problem have remained unknown. 

The present study aimed to investigate patients and caregivers-related risk factors that are related to psychiatric hospitalization of patients.

## Materials and Methods

*Participants*

Two hundred and thirty one adult patients (study subjects as the clients) and their caregivers, who were users of educational programs -provided by The Iranian Society Supporting Individuals with Schizophrenia (ISSIS) in Tehran- were eligible to be enrolled in the study and completed all measures. 

Those subjects who were users of alcohol and substance expect nicotine, or were diagnosed with brain organic syndrome, mental retardation; comorbid psychiatric and/or physical disorders were excluded. The majority of the subjects were middle-aged and unmarried males but we also recruited female consumers and the majority of caregivers were women especially elderly mothers. Written informed consent forms were obtained from the participants. The protocol of the study was approved by the Institutional Review Board of Shahid Beheshti University in Tehran, Iran in 2009.

***Measures***

*Patient measures*

All the clients met the fourth edition of the Diagnostic and Statistical Manual of Mental Disorders (DSM-IV) criteria for schizophrenia. Symptoms of schizophrenia were assessed by Scale for the Assessment of Positive Symptoms (SAPS) (16) and Scale for the Assessment of Negative Symptoms (SANS) (16). In the present study, adequate internal Cronbach’s alpha coefficients for positive and negative symptoms were 0.84 and 0.88, respectively. Basic life skills were assessed by the Kohlman Evaluation of Living Skills (KELS) (17). In this study, an acceptable internal Cronbach’s alpha coefficient was 0.70. 

*Caregiver measures*

Family knowledge about schizophrenia was assessed by knowledge questionnaire for caregivers. We used the modified version of this questionnaire which was developed by Khazailie (18). It consists of 31 true/false questions which covers the symptoms, treatment and family’s awareness and behavior toward the patient. 

The reliability of this questionnaire via test-retest within a week was 0.82. Cronbach’s alpha coefficient in the present study within a week was 0.74. The Family Experiences Interview Schedule (FEIS) was administered for care givers to assess family characteristics and burden (19). 

In this study, we administered a short version of this schedule including 41 questions and test-retest reliability and adequate Cronbach’s alpha coefficient (0.89) within a week (20) and in this study, highly reliable Cronbach’s alpha coefficient was 0.90. Social Support Scale (SSS) (21) was administered to assess the perceived social support among the caregivers. Adequate internal Cronbach’s alpha coefficient of this scale in the present study was 0.70. 

Family Assessment Device (FAD) devised by Epstein et al. (22) was administered to assess family function, structural and organizational characteristics of family and the patterns of relationship among family members in seven main aspects including problem solving, communication, roles, affective responsiveness, affective involvement, behavior control and general functioning. 

Reported adequate internal Cronbach’s alpha in study of Epstein et al. (22) ranged from 0.72-0.92 among the subscales. In this study, reliable Cronbach’s alpha ranged between 0.60-0.85. 

*Data analyses*

Logistic regression model analyses were performed using SPSS for Windows 16.0 (SPSS, Inc., Chicago, IL, USA) to explore the predictive contributions of various variables assessed with variable of referring clients to psychiatric hospitalization by their caregivers.

## Results

The caregivers’ age range was 19 to 84 years. Most of them were women (71.9%) with mean age of 61.5 years who were typically mothers (43.3%) while the rest of the caregivers were fathers (18.2%), sisters (14.3%), spouses (12.1%) and brothers (9.1%) or were other family members such as granddaughters (1.3%); all were living in the same houses with their patients. The subjects’ age range was 18 to 64 years. The majority of the clients were men (79.2%) while the rest were women (20.8%), with mean age of 43.1 years who were typically unmarried (67.1%) and unemployment (83.5%). The frequency of lifetime psychiatric hospitalization for schizophrenia was 5.2 times and average length of illness was 8.8 years (Table 1).

***Consumers-related analyses***

*Demographic and clinical characteristics*

The model showed 3 demographic variables i.e. older age (r = 0.057) as the strongest predictor, lower level of education (r = -0.460) and unemployment (r = -1.40) were predictors of referrals but other variables were not statistically significant predictors (Table 2).

**Table 1:** Demographic and clinical characteristics of the caregivers and their schizophrenic patients

**Variable**		**Consumers (n = 231)**	**Caregivers (n = 231)**
**Gender [n(%)]**	Male	65 (28.1)	183 (79.2)
	Female	166 (71.9)	48 (20.8)
**Age (Year)**		61.5 ± 59.1	43.1 ± 38.1
	> 12 years	116 (50.2)	109 (47.2)
**Education [n(%)]**	12 years	87 (37.6)	95 (41.1)
	< 12 years	28 (12.1)	27 (11.7)
**Marital status [n(%)]**	Currently married	154 (66.7)	37 (16.0)
	Never married	15 (6.5)	155 (67.1)
	Divorced	19 (8.2)	35 (15.2)
	Widow/widower	43 (18.6)	4 (1.7)
**Employment [n(%)]**	Currently employed	-	38 (16.5)
	Currently unemployed	-	193(83.5)
**Monthly income [n(%)]**	> $ 500	209 (90.5)	-
	Between $ 500-$750	12 (5.2)	-
	< 750	9 (3.9)	-
**Medical insurance [n(%)]**	Currently owns	-	181 (79.4)
	Currently does not own	-	50 (21.6)
**Family relation with consumer [n(%)]**	Mother	100 (43.3)	-
	Father	42 (18.2)	-
	Sister	33 (14.3)	-
	Spouse	28 (12.1)	-
	Brother	21 (9.1)	-
	Others	3 (1.3)	-
**Frequency of lifetime psychiatric ** **hospitalization**		-	0-25
**Number of lifetime psychiatric hospitalization**	(Year)	-	5.2 ± 6.2
**Frequency of length of illness **		-	2-44
**Length of illness (Year)**		-	8.8 ± 16.0

**Table 2.** Predictions of schizophrenic patients’ demographic and clinical variable in referral

**Variable**	**B**	**SD**	**Wald test**	**df**	**Significant level**	**EXP (B)**	**95% CI **	
							**Minimum**	**Maximum**
**Age**	0.057	0.027	4.33	1	0.037	1.05	1	1.1
**Gender**	0.019	0.369	0.003	1	0.960	1.01	0.47	2
**Education**	-0.460	0.142	10.46	1	0.001	0.631	0.47	0.83
**Employment status**	-1.40	0.452	9.66	1	0.002	0.245	0.10	0.59
**Number of psychiatric hospitalization**	0.005	0.002	0.094	1	0.760	0.999	0.99	1
**Length of illness**	0.009	0.032	0.083	1	0.773	1.009	0.98	1
**Fixed value**	-0.687	0.798	0.740	1	0.390	0.503		

***Symptom-related and basic life skills-related characteristics***

The model showed all variables i.e. greater severity of negative symptoms (r = 0.922) as the strongest predictor, greater severity of positive symptoms (r = 0.496) and greater deficiency in basic life skills (r = 0.705) were statistically predictors of referrals (Table 3).

***Family caregivers-related analyses***

*Family characteristics and burden*

Family characteristics and burden was another important part of the data analysis. The model showed experiencing greater objective burden by caregivers (r = 0.509) as the strongest predictor and lower knowledge of family about schizophrenia (r = -0.749) were significantly predictors of referral, while other variables were not statistically significant predictors (Table 4).

*Availability of supportive and health-related services*

Finally, availability of supportive and health-related services was explored. The model showed low perceived social support among caregivers (r = -0.491) as the strongest predictor and lack of medical insurance for consumers (r = -1.51) were significantly predictors of referral but other variables were not statistically significant predictors (Table 5).

## Discussion

Best to our knowledge, this is the first study in Iran that has been conducted on Iranian schizophrenic patients and caregivers-related risk factors associated with referral of clients to psychiatric hospitalization. This issue has important clinical and financial implication for Iran. As a developing country, Iran has limited psychiatric services and financial resources to provide hospitalization for its consumers and support their caregivers. Therefore, identifying factors associated with this problem could partly contribute to decreasing this burden on families and the community. 

**Table 3:** Predictions of schizophrenic patients’ symptom-related and basic life skills-related variables in referral

**Variable**	**B**	**SD**	**Wald statistic**	**df**	**Significance level**	**EXP (B)**	**95%**	
							**Minimum**	**Maximum**
**Negative symptoms**	0.922	0.302	9.34	1	0.002	2.51	1.39	4.54
**Positive symptoms**	0.496	0.191	6.72	1	0.010	1.64	1.12	2.39
**Basic life skills**	0.705	0.299	5.56	1	0.018	2.023	1.12	3.63
**Fixed value**	0.045	0.178	0.064	1	0.800	0.956		

**Table 4:** Predictions of caregivers’ characteristics and burden variables in referral

**Variable**	**B**	**SD**	**Wald statistic**	**df**	**Significance level**	**EXP (B)**	**95% CI **	
							**Minimum**	**Maximum**
**Problem-solving**	-0.263	0.240	1.190	1	0.274	0.769	0.48	1.23
**Communications**	0.203	0.236	0.745	1	0.388	1.220	0.77	1.94
**Roles**	-0.079	0.276	0.081	1	0.776	0.924	0.53	1.58
**Affective responsiveness**	0.068	0.258	0.070	1	0.791	1.070	0.64	1.77
**Affective involvement**	0.229	0.228	1.010	1	0.315	1.250	0.80	1.96
**Behavior control**	0.164	0.261	0.392	1	0.531	1.170	0.70	1.96
**General functioning**	-.0450	0.350	1.650	1	0.199	0.638	0.32	1.26
**Objective burden**	0.509	0.209	5.93	1	0.015	1.660	1.10	2.50
**Subjective burden**	0.200	0.237	0.709	1	0.400	1.210	0.76	1.94
**Family knowledge**	-0.749	0.194	14.81	1	0.001	0.473	0.32	0.69
**Fixed value**	0.083	0.158	0.277	1	0.590	1.080		

**Table 5:** Predictions of variables of supportive and health-related services in referral

**Variable**	**B**	**SD**	**Wald statistic**	**df**	**Significance level**	**EXP (B)**	**95% CI **	
							**Minimum**	**Maximum**
**Perceived social support**	-0.491	0.099	24.650	1	0.001	0.612	0.612	0.36
**Medical insurance**	-1.51	0.519	8.530	1	0.003	2.22	2.22	6.35
**Substitute caregiver**	-0.189	0.592	0.102	1	0.750	0.828	0.828	1.96
**Assistant for caregiver**	-0.129	0.591	0.047	1	0.828	0.879	0.879	1.69
**Supportive organization**	0.724	0.489	2.190	1	0.139	2.063	2.063	2.10
**Fixed value**	4.096	00.837	23.950	1	0.000	60.08		

We found out that older, less educated and unemployed patients were significantly at higher risk for referral, but contribution of older age was more important compared to the other two variables. It may be partly due to this issue that older age of patients was associated with long years of facing caregivers with schizophrenia as a significant stressor in their families which increased long-term emotional and practical burden of the care. Some parts of these findings were in accordance with other studies which demonstrated that older age of psychiatric patients increased the possibility of admission to psychiatric hospitals (23) and are in contrast with some studies that claim young age of schizophrenic patients is a burden for their caregivers (24) and is associated with readmission to psychiatric hospitals (25). 

The lack of adequate education and unemployment among clients to manage themselves were also likely to increase financial burden of care among the caregivers who were typically elderly women, especially mothers who were financially dependent or had limited financial resources to support themselves and their patient members at the same time.

These finding were in accordance with some studies that indicated unemployment among some of severely mental ill patients increased the number of frequent admission of psychiatric patients to psychiatric hospitals (23). About other demographic and clinical characteristics of the patients, the current study found little association with gender, the number of psychiatric hospitalization and duration of illness with referral. These findings were in agreement with some studies which indicated gender, the severity of illness such as number of hospitalization and length of illness do not act as burden for caregivers of individuals with schizophrenia (26). 

One of the aspects of our findings was the strong association between symptoms of patients in predicting referral especially the negative symptoms which were crucially important compared to the positive symptoms. It may be partly due to this issue that these variables were a stress-distress paradigm for caregivers especially when they realized low control over them which were likely to result in referral. These findings supported other research findings which indicated severity of positive and negative symptoms increased admission to psychiatric hospitals (27). 

We found deficiency in managing basic life skills served as a strong predictor in referral. Long-term responsibilities for handling domestic routines of the consumers sometime for years were likely to impose overall burden on their caregivers who had also responsibilities before other members of their families and reduced their motivations for household maintenance of the patients which subsequently facilitated referral which was in accordance with the study results of Chimeh et al. on the role of sufficient living skills of patients in imposing burden on their caregivers (28). 

Among variables associated with caregivers, it was found that those who reported experiencing greater objective burden were more likely to refer their patients. It may be partly due to this issue that behavioral problems among patients led to disrupted lives of their caregivers and were likely to prevent social contacts, leisure activities and handling domestic routine that were likely to result in financial and vocational difficulties for their caregivers and, pursued them to refer their patients to psychiatric hospitals to restore their peaceful family atmosphere. This finding was in accordance with some studies that suggested behavioral problems of mentally ill individuals disrupt lives of their caregivers and result in objective burden (29).

Moreover, less knowledge about schizophrenia among caregivers served as a significant predictor in referral. This finding may be the result of this fact that those caregivers who perceived schizophrenia as distress, did not know how to cope with it in critical conditions due to patients and reacted to disruptive behaviors and symptoms of patients in household care with negative emotions such as fear, anxiety, rejection, anger or shame, which were more likely to refer their patients to avoid these issues. Our study finding was in consistent with other studies that revealed lower knowledge leads in burden in caregivers (30).

Finally, among variables related to supportive and health-related services, an important finding was the fact that perception of low social support served as a significant predictor in referral compared to other variables in this section. Those caregivers who perceived less social support in managing their patients reported greater burden, more perceived stigma, social isolation and subsequently more referral. This is likely to be the result of realizing lower social supports among the caregivers. Realizing high social support could be associated with lower burden among care givers. This finding was in accordance with a study which found caregivers who had lower overall perceived social support or felt they were not getting enough support from other family members, experienced greater burden (26). 

One of the interesting findings in this part of the study was the role of medical insurance in referral. The fact that medical insurance reimburses at least a part of medical and health expenditures related to schizophrenia and partly compensates financial burdens of schizophrenia on family was reported as significant concern by many caregivers in this study. It might be due to this issue that those caregivers who were less able to manage financial pressures due to schizophrenia reported greater burden and more referral. This finding was in accordance with the study of Folsom et al. on homeless individuals treated for severely mental illness who found that lack of medical insurance served as a crucial risk factor associated with homelessness (31). 

We limited our sample to ISSIS with mainly male subjects and a number of female clients and caregivers which is subject to further studies with larger and more diverse sample populations particularly with female clients and other caregivers such as employed caregivers and relatives but our sample was representative of the group we studied and the findings extended Iranian literature on schizophrenia.

Although the study results did not establish causation but based on the findings, psychoeducation interventions must be developed to reduce referral in schizophrenia symptoms and lower the rate of need to inpatient services in Iran. Giving information about schizophrenia to families followed by developing community-based facilities for consumers and their families enhance their contribution to household maintenance while this is important to know that enhancing supportive professional and social network through tapering decreasing stigma and providing some assistant facilities for critical periods can lower the burden of care giving and reduce admission rate to psychiatric hospitalization. 
